# Deep learning-based anomaly-onset aware remaining useful life estimation of bearings

**DOI:** 10.7717/peerj-cs.795

**Published:** 2021-11-26

**Authors:** Pooja Vinayak Kamat, Rekha Sugandhi, Satish Kumar

**Affiliations:** 1 Symbiosis Institute of Technology, Symbiosis International (Deemed University), Pune, India; 2 Department of CSE and IT, MIT School of Engineering, MIT-ADT University, Pune, India; 3 Symbiosis Centre for Applied Artificial Intelligence, Symbiosis International (Deemed University), Pune, India

**Keywords:** Deep learning, Predictive maintenance, Anomaly detection, Remaining useful life, Bearing, LSTM, Autoencoder, K-means, clustering

## Abstract

Remaining Useful Life (RUL) estimation of rotating machinery based on their degradation data is vital for machine supervisors. Deep learning models are effective and popular methods for forecasting when rotating machinery such as bearings may malfunction and ultimately break down. During healthy functioning of the machinery, however, RUL is ill-defined. To address this issue, this study recommends using anomaly monitoring during both RUL estimator training and operation. Essential time-domain data is extracted from the raw bearing vibration data, and deep learning models are used to detect the onset of the anomaly. This further acts as a trigger for data-driven RUL estimation. The study employs an unsupervised clustering approach for anomaly trend analysis and a semi-supervised method for anomaly detection and RUL estimation. The novel combined deep learning-based anomaly-onset aware RUL estimation framework showed enhanced results on the benchmarked PRONOSTIA bearings dataset under non-varying operating conditions. The framework consisting of Autoencoder and Long Short Term Memory variants achieved an accuracy of over 90% in anomaly detection and RUL prediction. In the future, the framework can be deployed under varying operational situations using the transfer learning approach.

## Introduction

**Figure 1:**
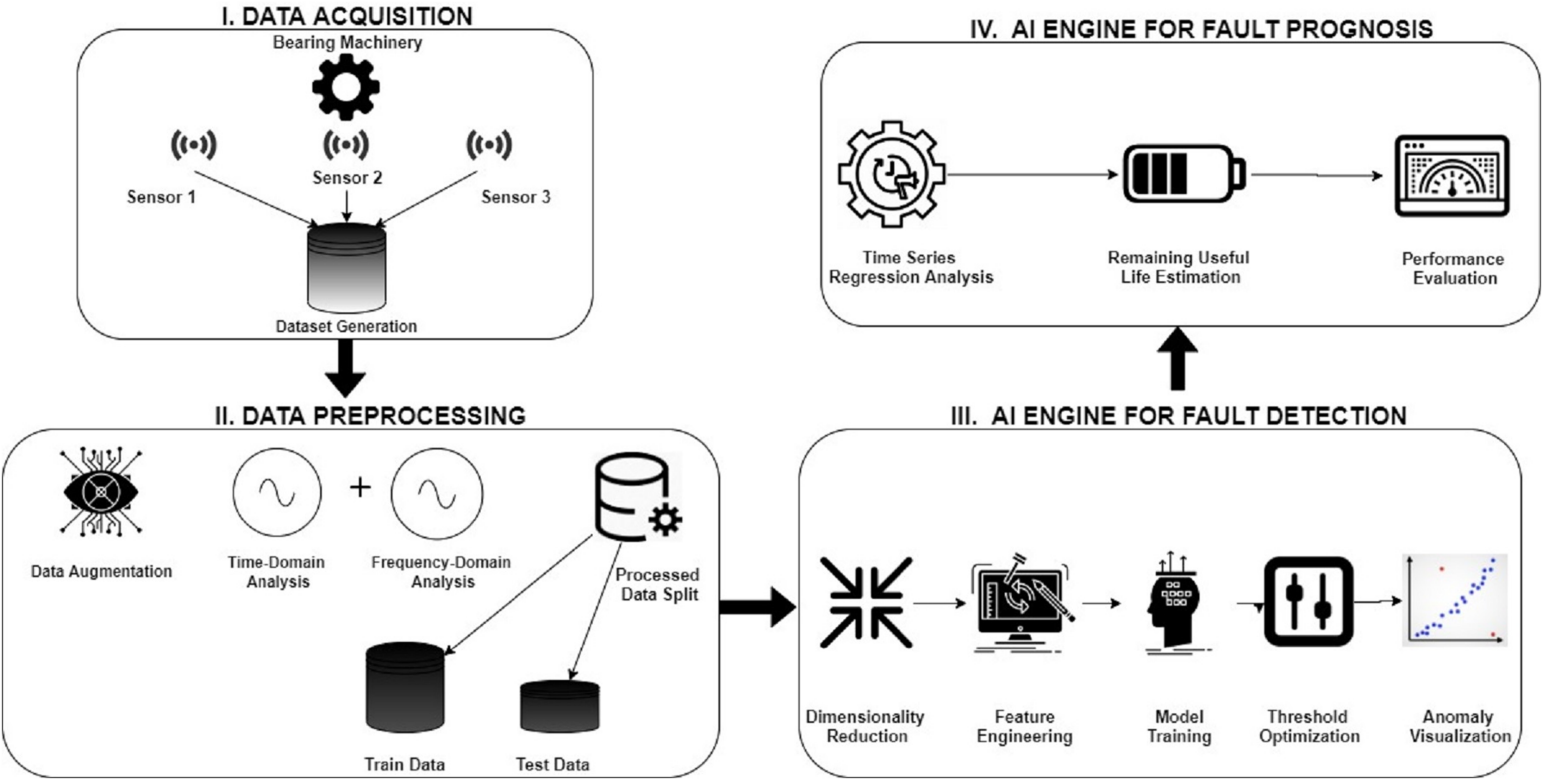
Predictive maintenance framework for bearing machinery.

Due to recent developments in telecommunications, sensor, and information technology, the availability of sensor data from industrial machines has increased, culminating in the fourth industrial revolution and smart manufacturing (Kurfess et al., 2020). Predictive maintenance, one of the numerous opportunities provided by the new industrial age, has garnered considerable attention in the recent decade because of its potential to increase production while lowering maintenance costs (Lee et al., 2015). Prognostics and Health management (PHM) aims to increase equipment dependability and availability while reducing maintenance expenses (Wu et al., 2018). Mainly, remaining useful life (RUL) is defined as the amount of time-related equipment has before it is defective (Agrawal et al., 2021). A rolling bearing is a vital component of rotating machinery, and its functioning has a significant influence on the machinery’s health (Wang, Tsui & Miao, 2017). As a result, diagnosing its running state is required to avert production loss or even casualties because of catastrophic issues. Rolling bearing RUL estimate accuracy ensures manufacturing safety and saves significant costs (Lee et al., 2014). A typical predictive maintenance framework for bearing machinery is shown in Fig. 1. The first step starts with acquiring condition monitoring data of the bearing machinery such as vibrations, temperature, sound, etc., using intelligent sensors such as accelerometers, thermal and acoustic sensors. The next step is to convert this raw signal data into a more primed dataset using signal processing techniques and FFT techniques such as time-domain, frequency-domain, and time-frequency domain analysis. The processed dataset is then split into training-test data for model training and validation. The third step is to apply the AI models for fault detection, which involves feature engineering techniques such as data denoising and feature selection. Suitable AI models are used, and model hyperparameters are optimized to improve the accuracy of anomaly detection. The final step is fault prognosis, whose aim is to do anomaly trend analysis and estimate the remaining useful life of the bearing machinery.

Currently, RUL-estimation is driven by two kinds of techniques-model-based and data-driven (Kara, 2021). To estimate the RUL of important physical components (such as bearing wear, inner-race faults, outer-race faults, etc.), model-based approaches (Jouin et al., 2016) use mathematical or physical models of degradation processes. It is challenging to correctly develop the deterioration model of composite components under complicated settings since it frequently demands much prior knowledge. The model-based techniques have the following challenges:

- Sequential Dependencies: The numerous machinery components interact in a myriad fashion, resulting in intricate temporal correlations between different sensors. Exploiting sensor readings to capture complicated machine operating characteristics with respect to time dependencies is crucial and necessary (Xu et al., 2020).
- Noisy Sensor Measurements: Sensor measurements are interfered with background environmental noise. Each sensor may have different noise bands, and capturing them in the model might be difficult (Luo et al., 2020).
- Health Degradation curve: In model-based systems, developing a health degradation curve based on various components requires high domain expertise and an enormous amount of historical data (Lin et al., 2020).

The variation in health degradation curves and physical characteristics across varied bearing systems poses challenges in developing an efficient model for RUL estimation. The goal of data-driven techniques (Zhao et al., 2019) is to transform sensor data into a stochastic or non-stochastic correlation model. It is not necessary to understand the process of deterioration; instead, adequate historical data is required. Compared to model-based and statistical techniques, deep learning (DL) is presently the dominating paradigm in RUL estimation (Sayyad et al., 2021). Complex models may be learned using deep learning algorithms using large amounts of training data. Deep learning algorithms widely used in RUL estimation include autoencoders (Xu et al., 2021), convolutional neural networks (CNN), CNN with attention mechanism (Tan & Teo, 2021; Wang et al., 2021), long-short term memory (Xia et al., 2021), gated recurrent unit (She & Jia, 2021), random forest (Chen et al., 2020) and support vector machines (Xue et al., 2020).

Selecting the reference truth RUL for the collected data is one of the most difficult challenges in developing a deep learning model for RUL estimation. RUL can be defined as a linearly descending function that tends to zero as the machine reaches failure. However, RUL-estimation is not accurately defined prior to degradation onset. Most research works handle this issue by establishing a maximum RUL level and assuming constant RUL beyond that level (Wu et al., 2021). Furthermore, the optimum RUL is an arbitrary decision, and the best value for different systems isn’t always the same (Zheng et al., 2017). Very little research work has been established to develop a common framework for detecting anomalies and predicting RUL from the onset of the anomaly. Most relevant bearing research investigations have focused on RUL estimations during the wear-out period (Javed et al., 2015; Patil et al., 2019). Most of the techniques do not mention the detection of bearing deterioration. Estimating the RUL when bearings are healthy is difficult, so identifying the start of wear is a good way to conduct fault forecasting. Most of these approaches require significant amounts of data to understand past examples to have excellent RUL estimates. As a result, when there isn’t enough data, these approaches can only be used to a limited extent. Furthermore, these approaches do not take into account the deterioration process. They have a hard time explaining the onset of the anomalies.

The primary motivation behind using a data-driven deep learning framework is its ability to automatically learn the correlation between the features of different bearing systems without extensive manual intervention.

The novelty of this research study is the design of a combined semi-supervised anomaly detection-RUL estimation technique using deep learning techniques that predict RUL only after the occurrence of the first anomaly. Thereby the framework is termed as Anomaly-Onset Aware RUL estimation framework. The mechanism of the proposed approach is as follows: at first, the time-domain features of the raw vibrational data are extracted, feature engineering is carried out to select the top features having the highest correlation with the target variable, next unsupervised clustering is carried out to analyze the anomaly trends in the curated data, anomalies are detected along with their timestamps using the Autoencoder-Long Short Term Memory (LSTM) model, and finally RUL prediction on the anomaly triggered data is achieved using the various variants of LSTM model. The entire methodology was applied on the popular run-to-failure dataset of PRONOSTIA bearings (Nectoux et al., 2012).

This study makes the following contributions:

- The application of unsupervised clustering approach using the k-means technique with Silhouette Coefficient for anomaly trend analysis.
- The development of a novel deep-learning based Anomaly-Onset Aware RUL (AOA-RUL) prediction framework for bearing machinery using selected time-domain extracted features based on probability ranking. The anomaly detector indicates the time stamp at which the first anomaly occurred. The anomaly detection is developed using the hybrid Autoencoder-LSTM technique.
- Further, the RUL regressor is trained and evaluated on degradation onset data alerted by the anomaly detector. The Anomaly-Onset Aware RUL prediction’s primary goal is to prevent RUL estimation during healthy operations when the RUL estimation is not necessary. Various LSTM variants are used to implement the RUL regressor, and a comparative analysis is presented.

This technique improves RUL estimate accuracy while lowering computing cost by avoiding superfluous calculation prior to degradation onset, *i.e*., from the observed degradation onset to the entire failure timestamp of the bearing data. The dataset and methodology are described in the next section.

## Materials & Methods

### Dataset description

The PRONOSTIA dataset (Nectoux et al., 2012) utilized in this study refers to the failure of seven bearings when running at 1,800 rpm and 4,000 N. These bearings are operated until they fail, ensuring that no flaws are seeded into the bearings. As a result, the bearings may fail due to any fault, *i.e*., inner race fault, outer race fault, cage fault, etc. The bearing vibration signals are captured in the horizontal and vertical directions has two accelerometers, X and Y. Furthermore, failure prediction is complicated by less training data and considerable variability (8,710–28,030 s) in trial length. As a result, the challenge addressed in this study is to decrease noise in the data due to the presence of various failure behaviors and to create an accurate RUL forecast. Deep learning models are very effective in reducing noise from the data due to their excellent feature extraction capabilities (Kundu, Chopra & Lad, 2019). Table 1 describes the final failure time of each of the seven bearings.

**Table 1:** Actual RUL values for Bearings 1 to 7.

Bearing_No	1	2	3	4	5	6	7
**Time in seconds**	28,030	8710	23,750	14,280	24,630	24,480	22,590

### Anomaly-onset aware RUL prediction framework

**Figure 2:**
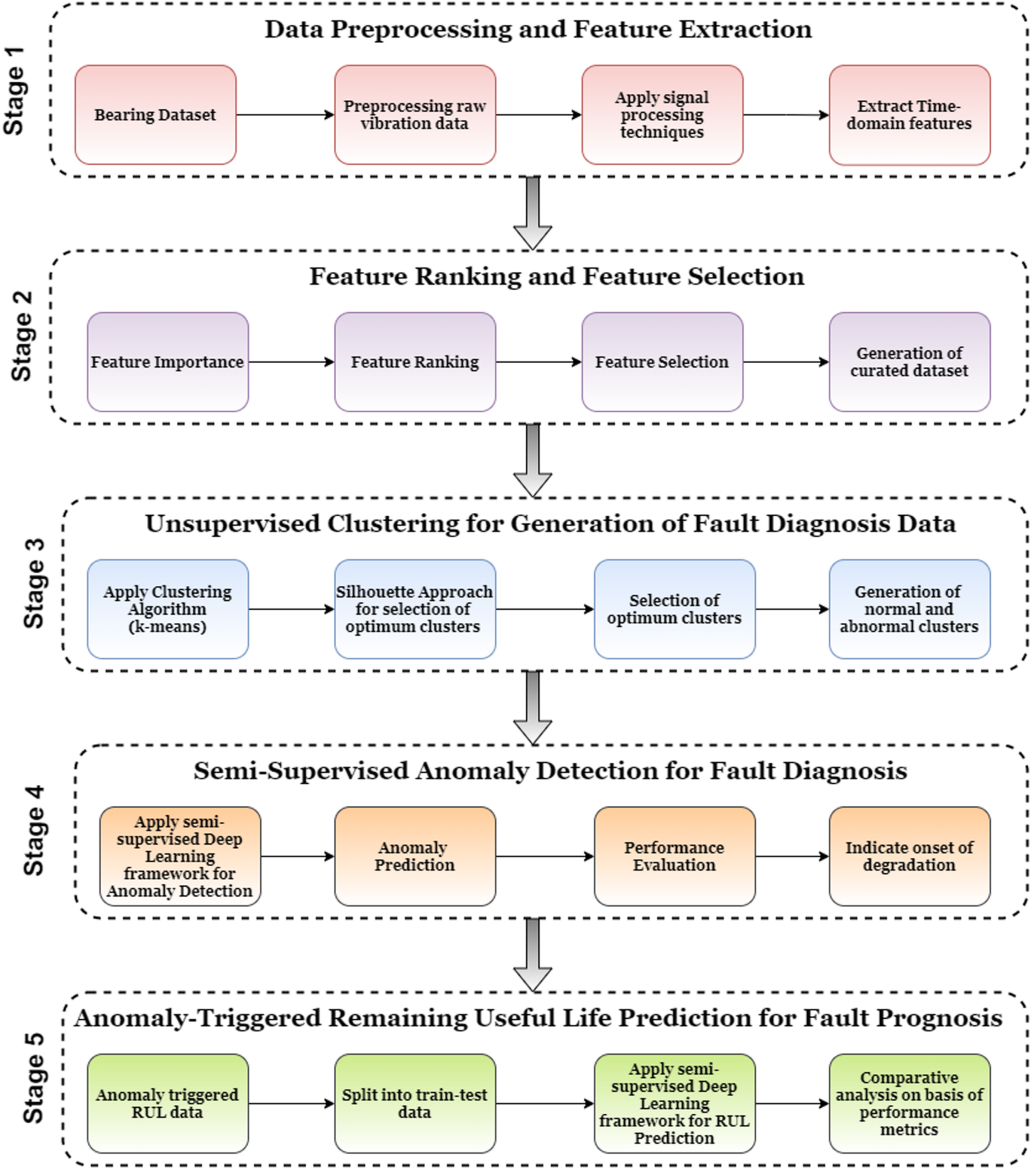
Anomaly-Onset Aware RUL prediction framework.

Figure 2 describes the proposed Anomaly-Onset Aware RUL Prediction framework. The novelty of this framework is the development of a combined technique for fault detection and fault prognosis. The framework uses unsupervised clustering techniques for anomaly trend analysis and supervised deep learning techniques to identify anomaly onset and RUL estimation. The framework consists of five stages: (i) Data Preprocessing and Feature Extraction; (ii) Feature Ranking and Feature Selection; (iii) Unsupervised Clustering for generation of Fault Diagnosis Data; (iv) Semi-Supervised Anomaly Detection for Fault Diagnosis and (v) Anomaly-triggered RUL prediction for Fault Prognosis. Each of the stages is described in detail as follows:

### Stage 1: Data preprocessing and feature extraction

The vibration data is very significant to forecast the life of a bearing. As the bearing approaches failure, the amplitude and distribution of the vibration signal varies. A single vibration signal represents the number of data points captured in a specific period. For example, in this scenario, 2,560 data points are captured after a 10-second interval and are treated as one signal. The total amplitude level of these 2,560 data points will alter as the fault severity increases. In addition, the signal’s mean and standard deviation, which indicate the signal distribution, will be altered. The raw vibration data collected from the sensors, on the other hand, may not be adequate to indicate bearing deterioration (Kundu, Darpe & Kulkarni, 2019). To get meaningful information regarding component deterioration, features are often derived from natural vibration data (Caesarendra & Tjahjowidodo, 2017). According to prior studies, the significant time-domain parameters that define bearing failures include RMS, peak, kurtosis, skewness, and crest factor obtained from raw vibration signals (Kundu, Chopra & Lad, 2019; Li, Sopon & He, 2009; Lim et al., 2020). Around 21 features, including statistical parameters as well for accelerometers X and Y were extracted from the raw vibration data captured. Table 2 represents some of the major time-domain features used in this study. Here, x_i_ is a signal series for i = 1, 2, 3… N, and N is the number of data points.

**Table 2:** Time-domain extracted features.

Sr No.	Time-domain feature	Description	Formula
1.	*RMS (Root Mean Square)*	This value represents the vibration signal's energy content. As the fault develops, the RMS value steadily rises.	$RMS = \sqrt {1/N(\mathop \sum \limits_{i = 1}^N x_i^2}$)
2.	*Variance, standard deviation, and other statistical parameters*	The dispersion of a signal around its reference mean value is measured by variance. The standard deviation is a statistical term that measures how much a signal varies. Other statistical parameters such as mean, median, etc provide additional information about the signal.	$Variance = \displaystyle{{\mathop \sum \nolimits_{i = 1}^N {{\left( {{x_i} - m} \right)}^2}} \over {\left( {N - 1} \right){\sigma ^2}}}$ $Std.\; Deviation = \displaystyle{1 \over N}{\left( {{X_i} - \bar X} \right)^2}$
3.	*Kurtosis and kurtosis coefficient*	The kurtosis measures how thick or heavy the tails of the data's probability distribution are. The kurtosis of a bearing vibration signal is an essential indication that can reveal the bearing's operational status.	${K_u} = \displaystyle{{\mathop \sum \nolimits_{i = 1}^N {{\left( {{x_i} - m} \right)}^2}} \over {\left( {N - 1} \right){\sigma ^2}}}$
4.	*Skewness and skewness coefficient*	Skewness is a probability density function that measures the asymmetry of a vibration signal.	${S_k} = \displaystyle{{\mathop \sum \nolimits_{i = 1}^N {{\left( {{x_i} - m} \right)}^3}} \over {\left( {N - 1} \right){\sigma ^3}}}$
5.	*Crest Factor*	A waveform's crest factor indicates the severity of its peaks. The crest factor identifies signal pattern variations caused by impulsive vibration sources such as a crack on the bearing's outer race.	$crest\; Factor = \displaystyle{{max\left| {{x_i}} \right|} \over {\sqrt {\displaystyle{1 \over N}\mathop \sum \nolimits_{i = 1}^N x_i^2} }}$
6.	*Entropy*	The variability and unpredictability of collected vibration data are calculated using entropy, e(p).	$e\left( p \right) = \mathop \sum \limits_{i = 1}^n p\left( {{z_i}} \right){\log _2}p\left( {{z_i}} \right)$
7.	*Peak value*	The signal's most significant deviation from zero, or equilibrium, is represented by the peak. The distance between a negative peak and a positive peak is measured in peak-to-peak amplitude. Variations in the PEAK estimate of vibration signals show progress in the signal due to the occurrence of bearing defects.	$Peak\; value\; \left( {Vpeak} \right) = max\left| {{x_i}} \right|$
8.	*Shape Factor*	It is the ratio of the RMS signal estimation to the overall signal estimation.	$shape\; factor = \displaystyle{{RMS\; value} \over {\displaystyle{1 \over N}\mathop \sum \nolimits_{i = 1}^N \left( {{{\bar X}_i}} \right)}}$
9.	*Clearance Factor*	It's the ratio of a signal's peak value to the square of the normal of the outright value signals' square foundation.	$clearance\; factor = \displaystyle{{peak\; value} \over {\displaystyle{1 \over N}\mathop \sum \nolimits_{i = 1}^N {{\left( {\overline {{X_i}} } \right)}^2}}}$
10.	*Impulse Factor*	It is the ratio of the signal's peak value to the normal of the signal's unambiguous estimation.	$impulse\; factor = \displaystyle{{peak\; value} \over {\displaystyle{1 \over N}\mathop \sum \nolimits_{i = 1}^N {{\left( {\left| {\bar X} \right|} \right)}^2}}}$

### Stage 2: Feature ranking and feature selection

At the end of stage 1, 22 time-domain features each for accelerometer X and Y were extracted. Exploratory data analysis on the extracted data was carried out to get more insights. Histogram plots representing the frequency distribution of each feature were plotted along with the correlation map. These graphs help to visualize the correlations between all the features in the dataset. The extracted features reflect bearing deterioration from various perspectives. However, if all of these variables are used as model input parameters, the model may become overfitted. Overfitting occurs when a model’s performance is good during training but dramatically worsens during testing. Hence selecting optimum features is essential. Feature selection is a method for identifying key equipment attributes and removing those that contribute little to the model’s output or goal variables. A suitable feature selection method increases the model’s prediction accuracy and performance substantially (Cheng et al., 2020; Lee & Wen, 2020). The following feature selection strategies were utilized in this study. Each feature would be given a feature ranking/importance based on the below methods. The term “feature importance” refers to a set of approaches for providing scores to input characteristics in a predictive model, indicating the relative significance of each information when generating a prediction. The following feature ranking techniques were used in this study.

- **Linear Regressor:** The primary objective of regression analysis is to develop an equation that can predict values for some predictor variables for all members of a population given explanatory variables. Linear regressor makes use of recursive elimination function for feature selection (Chen et al., 2017). RFE is widely used because it is simple to set up. It successfully identifies which features (columns) in a training dataset are more significant in predicting the target variable. The following is an example of a linear regression line equation:
(1) $$y = a + bX$$
  Here $X$ is the independent variable, which is displayed on the x-axis, and $y$ is the dependent variable, which is shown on the y-axis. The intercept (the value of y when x = 0) is $a$, while the slope of the line is $b$.
- **Random Forest Regressor:** Many decision trees make up a random forest. Every node in the decision trees is a feature qualifier to divide the dataset into two groups with similar response values. Impurity is the criterion by which the (locally) best condition is selected. As a result, it is possible to calculate how much each feature reduces the weighted impurity in a tree while training it. The impurity decrease from each feature in a forest may be averaged, and the features can be ranked based on this metric. High-ranking features can then be selected for optimal model performance (Ubing et al., 2019). On a decision tree, the significance of each feature is computed as follows:
(2) $$f{i_i} = \displaystyle{{\mathop \sum \nolimits_{j:node\; j\; splits\; on\; feature\; i} n{i_j}} \over {\mathop \sum \nolimits_{k\ \in\ all\; nodes} n{i_k}\; }}$$
  where $fi{\rm \; }sub\left( i \right) = {\rm \; }$feature i’s importance
$ni{\rm \; }sub\left( j \right){\rm \; } =$ node j’s importance
  By dividing by the total of all feature importance values, they may be normalized to a number between 0 and 1:
(3) $$norm\; f{i_i} = \displaystyle{{f{i_i}} \over {\mathop \sum \nolimits_{j\ \in\ all\; features} f{i_j}\; }}$$
  At the Random Forest stage, the average of all the trees is the most important attribute. The total number of trees is divided by the sum of the feature’s importance rating on each tree:
(4) $$RF\; f{i_i} = \displaystyle{{\mathop \sum \nolimits_{j\ \in\ all\; trees\; } norm\; f{i_j}} \over T}$$
  where $RF{f_i}{\rm \; }sub\left( i \right) =$ feature i ’s importance calculated from all the trees in the Random Forest.
- **Mutual Info Regressor:** Mutual information is calculated for fixed categories in a classification task or a continuous target variable in regression situations. The entropy of the variables is used to calculate mutual information. Mutual information (MI) between two random variables is a non-negative number that indicates how dependent the variables are on each other. By rating their mutual information with the target variable, we may choose the features from the feature space. The advantage of mutual information over the different statistical techniques is that it can handle non-linear relationships between features and target variables (Hashemi, Dowlatshahi & Nezamabadi-pour, 2021). Formally, the mutual information between two random variables X and Y is as follows:
(5) $$I\left( {X;Y} \right) = H\left( X \right) - H\left( {X|\; Y} \right)$$
  where $I\left( {X;Y} \right)$ represents mutual information between X and Y, $H\left( X \right)$ represents entropy for $X$ and $H\left( {X|{\rm \; }Y} \right)$ represents conditional entropy for $X$ given $Y.$

### Stage 3: Unsupervised clustering for generation of fault diagnosis data

Once desired features are selected, the next step is to perform clustering. The PRONOSTIA dataset consisted of the run to failure data of many bearings till degradation (Nectoux et al., 2012). During the lifetime of the bearings, faults could have occurred at any stage resulting in abnormal vibrations. However, these abnormalities were not annotated in the dataset. Hence performing unsupervised clustering at this stage is crucial. Clustering is an unsupervised learning method that can be used to analyze the selected features from Step 2. Clustering generally uses specified criteria to put representative samples into the same cluster and split disparate data into multiple clusters (Pu et al., 2021). The current work used K-means clustering, which is one of several clustering methods (Shorewala, 2021).

K-means clustering technique was used to form normal and abnormal clusters. An optimum number of clusters can be determined with the help of the Silhouette coefficient (Tambunan et al., 2020). The difference between within-cluster tightness and separation from the remainder is calculated using the silhouette coefficient. The Silhouette Coefficient, often known as the Silhouette score, is a measure for determining how successful a clustering approach is. Its value is between –1 and 1. Here,

1: Indicates that clusters are well separated and distinct from one another.

0 indicates that clusters are unrelated or that the distance between clusters is not significant.

–1: Clusters have been allocated incorrectly.

Mathematically Silhouette Coefficient (Sc) is calculated as below (Silhouette Coefficient, n.d.):

(6) $${S_c} = \displaystyle{{b - a} \over {max\left( {a,b} \right)}}$$

where it consists of the following two elements:

- The average distance between a sample and the rest of the samples in the same cluster a
- The average distance between a sample and the neighboring cluster b’s samples

K means algorithm with Silhouette coefficient is explained in Table 3 below:

**Table 3:** k-means algorithm with Silhouette Coefficient.

*Step 1:*	*Initialize with “k” number of clusters*
*Step 2:*	*Determine the centroid for each of the k clusters*.
*Step 3:*	*For each sample, assign it to the closest cluster centroid*
*Step 4:*	*Once each sample has been assigned, calculate the updated centroid for each cluster using the samples that have been allotted to that cluster. Cluster centroids tend to gravitate toward dense clusters*.
*Step 5*	*Compute the Silhouette coefficient (SC) for determining an optimal number of clusters. Reconfigure clusters and cluster centroids on the basis of SC value*
*Step 6:*	*If the new centroids differ significantly from the old centroids, the centroids have shifted. When the new and old centroids are the same, the method ends, ensuring that further iterations do not change the centroids*.

Unsupervised clustering is extremely helpful in the pattern mining of data when the data is in unlabeled form (Murugan, Sumithra & Murugappan, 2021). K-Means algorithm helped in clustering the vibration data into labeled categories of normal and abnormal. However, clustering alone can’t help to identify anomalous vibration signals over a sequence of time (Basora et al., 2021). In such a scenario, semi-supervised deep learning algorithms help predict threshold-based anomalous data along with the timestamp at which the first anomaly occurs.

### Stage 4: Semi-supervised anomaly detection for fault diagnosis

The task of discovering uncommon instances that stand out from the typical data is referred to as anomaly detection. Outliers or anomalous samples are of greater interest in various applications than normal samples. The detection of anomalies is the first and most crucial stage in any predictive maintenance effort. Anomaly detection can aid in machine health monitoring. Identifying the timestamp at when the first anomaly occurred can offer further information about the machinery’s remaining useful life (RUL). In this step 4, the hybrid Autoencoder-Long Short Term Memory (AE_LSTM) model is used for anomaly detection. In the case of Supervised Anomaly Detection, there is a difficulty with the binary categorization of “normal” and “abnormal” cases, and all instance labels should be known ahead of time. Class labels are heavily skewed toward the typical class, with anomalies appearing only sporadically. Unsupervised or unsupervised models treat the occurrences that do not fit the majority as anomalies, learn a pattern using partially labeled normal data, and finally scale anomaly likelihood based on the difference between an unknown pattern and the known normal pattern. Autoencoders belong to such a category of semi-supervised models, which flag an anomaly based on reconstruction loss. Since loss doesn’t have any labels, autoencoders are most suitable for anomaly detection tasks.

Further, most bearing fault data is sequential in character and is gathered over a longer period of time. Because the length of the input sequence might change, predicting over a longer sequence is challenging. The observation’s time-based ordering might make it difficult to evaluate only the essential characteristics for usage as input to supervised deep learning models. LSTM based recurrent neural models are apt in capturing temporal dependencies in data.

The hybrid Autoencoder-LSTM model’s core principle is to train it on both normal and abnormal clusters by creating a feature vector of all the selected features. It’s then encoded into an LSTM feature array with samples, features, and an n-timestep window. The sliding window will move over the whole sample space, and the encoder model will learn from the temporal data’s latent representations. The model will attempt to recreate the original data on the decoder side. At each stage, the reconstruction loss, or mean absolute error (mae), is computed. The reconstruction loss is very large in anomalous samples, and the sample can be flagged as an anomaly. The Autoencoder part of this model effectively reduces dimensionality, thereby capturing only latent informative features of the vibration signals during reconstruction. The LSTM model captures the sequential bearing degradation over a period of time.

Assume $F$ is the input feature vector of the bearings consisting of all the selected time-domain features ${f_1},{f_2}, \ldots .{f_n}$ for a particular timestamp t. At the encoder end, the feature vector is converted to a single LSTM feature array, and many such LSTM feature arrays are created for corresponding time values.

(7) $$F = \left\{ {{f_1},{f_2}, \ldots ,{f_n}} \right\};\; {f_i} \in original\; features\;

Next, the LSTM feature vector of the original sequence is encoded into latent space L, which is composed of a single hidden layer H. Multiple hidden layers with optimized weights can be further added.

(8) $$L = \left\{ {{l_1},{l_2}, \ldots ,{l_n}} \right\};\; {l_i} \in latent\; features\;$$

The decoder receives the latent LSTM feature vectors as input. R represents the reconstructed output at the decoder end, the reconstructed version of the original input F. The decoder tries to recreate all of the bearing system’s multivariate data that has been input. The LSTM input gate will capture the bearing information that has to be supplied to the autoencoder model. The forget gate decides which bearing information from the current cell state can be ignored, and the output gate displays the output data.

(9) $$R = \left\{ {{r_1},{r_2}, \ldots ,{r_n}} \right\};\; {r_i} \in reconstructed\; features\;$$

The anomaly score is the difference between the original input variable sequence F and the reconstructed output O determined at each step. Using the following loss function, the reconstruction error is attempted to be minimized during autoencoder training for higher model accuracy:

(10) $$\left( {loss\left( {F,R} \right)} \right) = 1/2\mathop \sum \limits_{i = 1}^n \parallel {f_i}\; - \; {r_i}{\parallel ^2}\;$$

**Figure 3:**
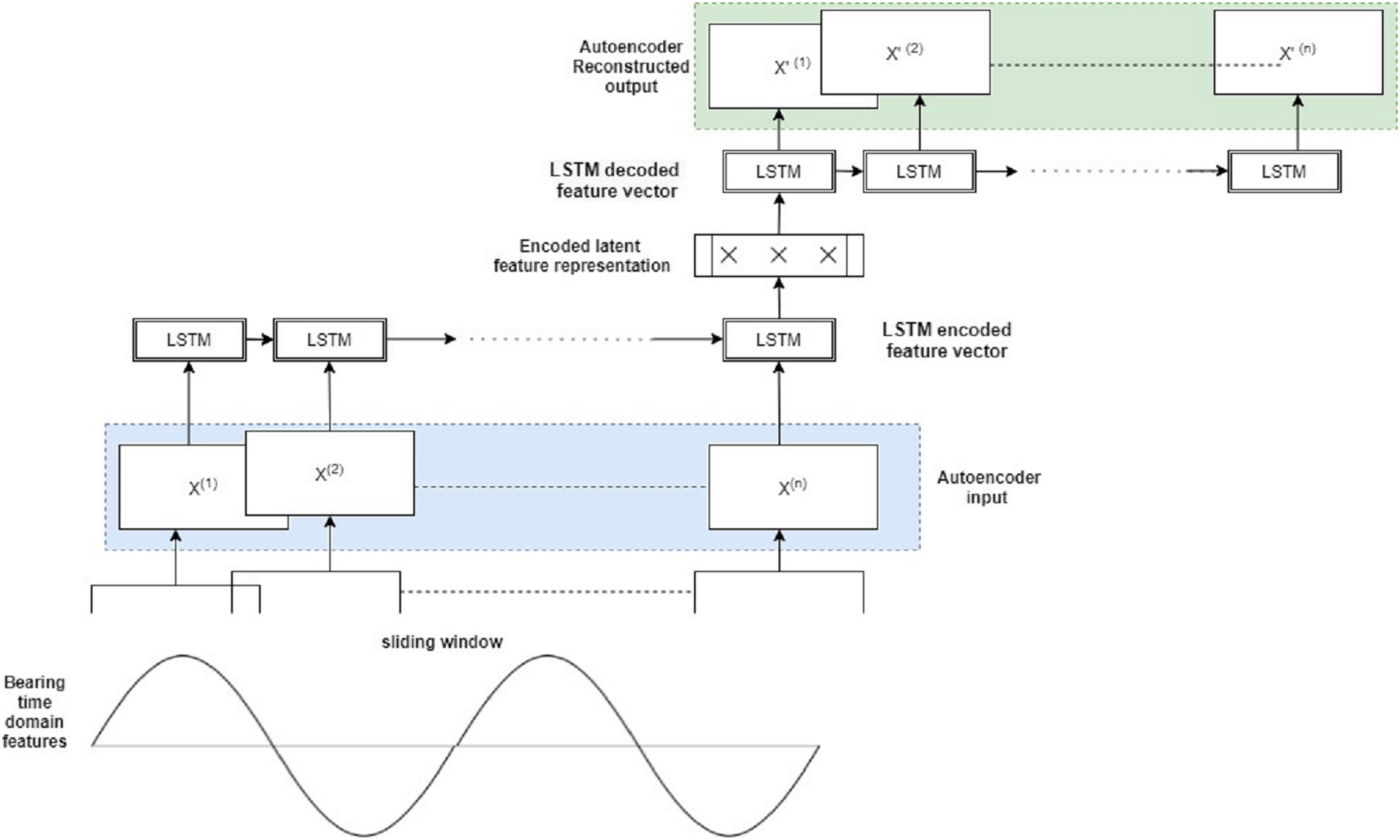
Proposed hybrid autoencoder-LSTM model for anomaly detection.

To establish the optimal threshold, a probability distribution map of all losses is plotted. The first timestamp at which the anomaly score exceeds the set threshold is finally labeled as the occurrence of the first anomaly. This step provides two important information for each of the seven bearings: (i) Anomalies/abnormal vibration signals (ii) Timestamp of the first anomaly occurrence. Both this information is extremely useful for the final step of this framework, *i.e*. Remaining Useful Life estimation. Fig. 3 represents the hybrid Autoencoder-LSTM model used for anomaly detection in this study.

### Stage 5: Anomaly-triggered remaining useful life prediction for fault prognosis

In the Predictive Maintenance paradigm, this is the final and most important phase. Estimating the remaining usable life (RUL) of industrial machinery based on deterioration data is essential for different sectors. Step 4 of Anomaly Detection assisted us in identifying the occurrence timestamp of the first anomaly. The Anomaly-Onset Aware RUL’s major goal is to prevent RUL estimation in healthy operations when the RUL is ill-defined. After the beginning of degradation, marked/flagged by the anomaly detector, anomaly onset aware RUL trains and tests RUL estimators. The major goal of the anomaly detection phase is to figure out when the system starts to degrade. The RUL estimate block is exclusively trained on active degradation, that is, from the detection of degradation through the total failure of the training samples. Various variants of LSTM models were used to implement the Anomaly-Onset Aware RUL framework.

Unlike the rest of the supervised learning techniques, sequence prediction or forecasting is unique and difficult. The sequence puts an order on the data that must be preserved while training models or providing predictions. Remaining Useful Life (RUL) prediction is a kind of regression-based sequential prediction wherein, based on previous condition monitoring data; we need to predict the usable life of the machinery. In the case of PRONOSTIA bearings, the selected time-domain features extracted over a period can help us predict the RUL of that particular bearing. The Long Short-Term Model (LSTM) is a recurrent Neural Network built particularly for sequential input. A Recurrent Neural Network may be thought of as a feedforward Multilayer Perceptron network with loops added to it. The recurrent connections provide the network’s “memory” or state, allowing it to learn and harness the ordered pattern of input sequences. The internal memory displays output based on the current context seen in the sequence rather than on the network’s provided input. In certain ways, this capability allows neural networks and deep learning to predict sequences.

LSTMs employ a set of ‘gates’ that regulate how information in a sequence of data enters, is stored, and exits the network. A typical LSTM has three gates: an input gate, a forget gate and an output gate. These gates are neural networks and may be thought of as filters. The first gate is the ‘forget gate’, which, when given the prior concealed state and fresh input data, selects the parts of the cell state for a particular timestamp t (long-term memory of the network) that are meaningful. The second gate is the input gate which, given the prior hidden state and new input data, this gate aims to determine what new data should be added to the network’s long-term memory (cell state) at that timestamp t. The third gate is the output gate, which is used to activate the LSTM block’s final output at timestamp t. The LSTM equations are given below:

(11) $${i_t} = \sigma \left( {{w_i}\left[ {{h_{t - 1}},{x_t}} \right] + \; {b_i}} \right)$$

(12) $${f_t} = \sigma \left( {{w_f}\left[ {{h_{t - 1}},{x_t}} \right] + \; {b_f}} \right)$$

(13) $${o_t} = \sigma \left( {{w_o}\left[ {{h_{t - 1}},{x_t}} \right] + \; {b_o}} \right)$$

**Figure 4:**
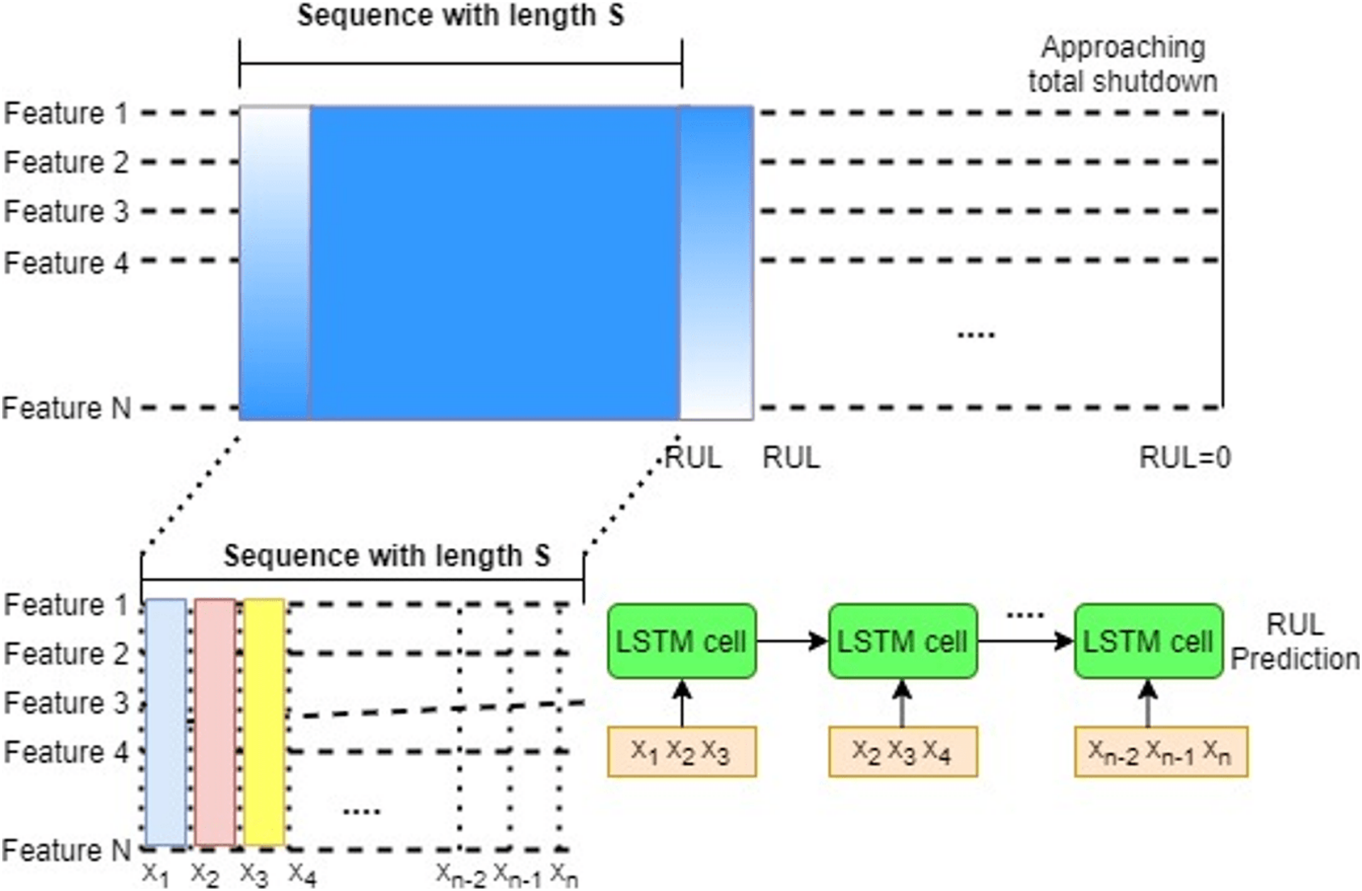
LSTM sliding window approach for supervised RUL prediction (A), Each feature is input into an LSTM network in a sequential order (B).

RUL prediction is a time series problem wherein the goal of RUL estimate tasks is to forecast the corresponding RUL at a given time. The time series problem is redefined as a supervised regression problem using the sliding window approach to train DL models, as seen in Fig. 4. Time series data is divided into continuous, fixed-length segments, each of which is labeled (the RUL). Simple RNNs were used in the early research to forecast the RUL (Heimes, 2008). LSTM models (Wu et al., 2018) consume the data sequentially at each point in time to solve the challenge of learning long-term temporal dependencies in RNN.

The sliding window approach can give more context and improve feature extraction (Zhou et al., 2021). In this scenario, all of the variables in the window are connected to an LSTM layer. Because the fully connected layer of the LSTM (FCLSTM) requires one-dimensional input for each time step, spatial and feature structure must be anticipated. Furthermore, the model complexity (number of weights) rises linearly with window size, which may be addressed with convolutional LSTMs in some cases (Zhou et al., 2021).

RUL estimate is formally viewed as a sequence to solve a problem. For a S-length sequence time series, $X = ({x_s}{\rm |}s = 1,2, \ldots ,S{\rm )}$ with ${x_{s{\rm \; }}} \in {\rm \; }{R^{n{\rm \; }x{\rm \; }m}}$. Here $n$ is the number of sensors, and $m$ is the number of instances per cycle. The goal of RUL estimation is to predict the corresponding RUL estimate ${y_s}$ where ${y_s} = f\left( {{x_S}|s = 1,2, \ldots ,S{\rm \; }} \right)$. Here ${x_s}$ denotes all samples at time stamp $s$. Using the sliding window approach, this equation can be modified as follows ${y_S} = f\left( {x_s^w|s = w,...,S{\rm \; }} \right)$. Here $w$ is the sliding window size. The feature vector $x_s^w$ consists of all samples in the time window $x_s^w = {x_{s - w + 1}}, \ldots ,{x_s}$ . The sequence length specifies how previous data is employed in any model, whereas the window size reflects the intricacy of dynamic characteristics across time. Both parameters should be explored while improving the model since they have a large impact on its performance.

The following variants of LSTM were used in this study.

- **Vanilla LSTM**: The Vanilla LSTM is a basic LSTM configuration. Vanilla LSTM is the name given to it in this to distinguish it from deeper LSTMs and the suite of more complex configurations. An LSTM model with a single hidden layer of LSTM units and a prediction layer is called a Vanilla LSTM (Xu et al., 2020). If we add multiple LSTM hidden layers, the architecture can be converted into a stacked-LSTM model.
- **Bi-Directional LSTM**: By walking through input time steps in both the forward and backward directions, bidirectional LSTMs address the challenge of getting the most out of the input sequence. This model is implemented by cloning the first recurrent layer of the network such that two layers are now side-by-side, then feeding the input sequence to the first layer and a reversed duplicate of the input sequence to the second layer (Wang et al., 2019).
- **Conv-LSTM**: In the Conv-LSTM architecture, Convolutional Neural Network (CNN) layers for feature extraction on input data are combined with LSTMs to give sequence prediction. The ConvLSTM is a kind of LSTM similar to the CNN-LSTM in that each LSTM unit contains a convolutional reading of the input. The ConvLSTM was created to read two-dimensional spatial-temporal data, but it may also be used to forecast multivariate time series (Li et al., 2021).
- **Encoder-Decoder LSTM**: Sequence-to-sequence prediction issues (seq2seq) are a more difficult form of problem in which a sequence is given as input and a sequence prediction is required as output. The Encoder-Decoder is made up of two sub-models: one that reads the input sequence and encodes it into a fixed-length vector, and the other that decodes the fixed-length vector and predicts the sequence (Liu et al., 2021). The encoder is often a Vanilla LSTM model, although alternative encoder models such as Stacked, Bidirectional, and CNN models can also be utilized. This model may be used to forecast multi-step time series.

**Figure 5:**
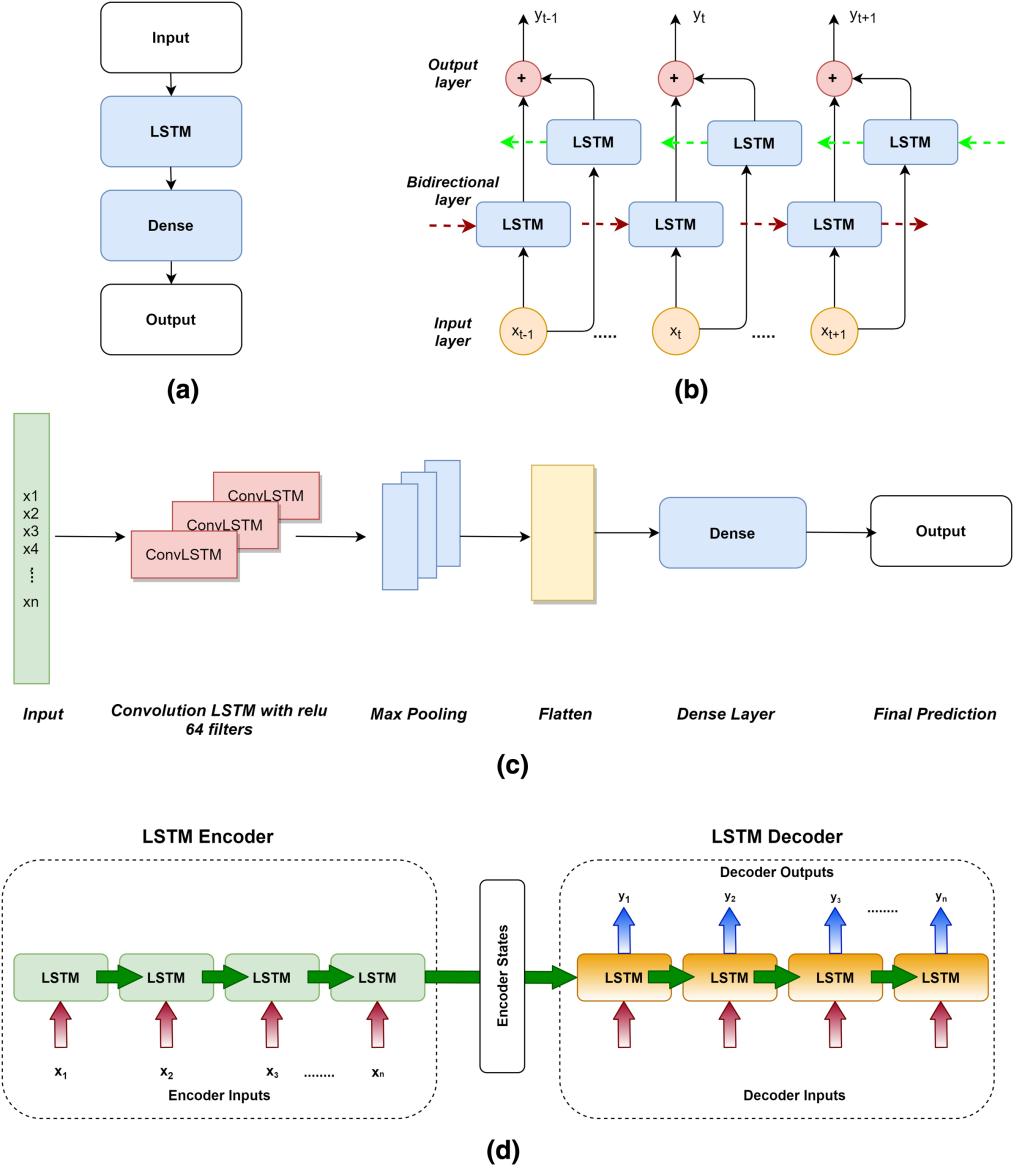
LSTM variants used for RUL prediction. (A) Vanilla LSTM. (B) Bidirectional LSTM. (C) ConvLSTM. (D) Encoder-Decoder LSTM.

All the four variants of LSTM used in this study are depicted in Fig. 5. (a) Vanilla LSTM, (b) Bidirectional LSTM, (c) ConvLSTM and (d) Encoder Decoder LSTM.

## Results

The Anomaly-Onset Aware RUL estimation framework is applied to Bearing 1–7 datasets. The authors now present the experimental results in this section:

### Feature extraction

Time-domain feature extraction was carried out on the vibration data collected *via* accelerometers X and Y. The following 21 time-domain features each were extracted on both the accelerometers at a particular timestamp: Mean squared error (mse), root mean square error (rms), crest factor, max_absolute_value, min_absolute_value, mean, median, mode, variance, standard deviation, peak2peak, entropy, skewness, skewness_coefficient, kurtosis, kurtosis_coefficient, change_coefficient, wave_factor, peak_factor, impulse_factor, and clearance_factor.

**Figure 6:**
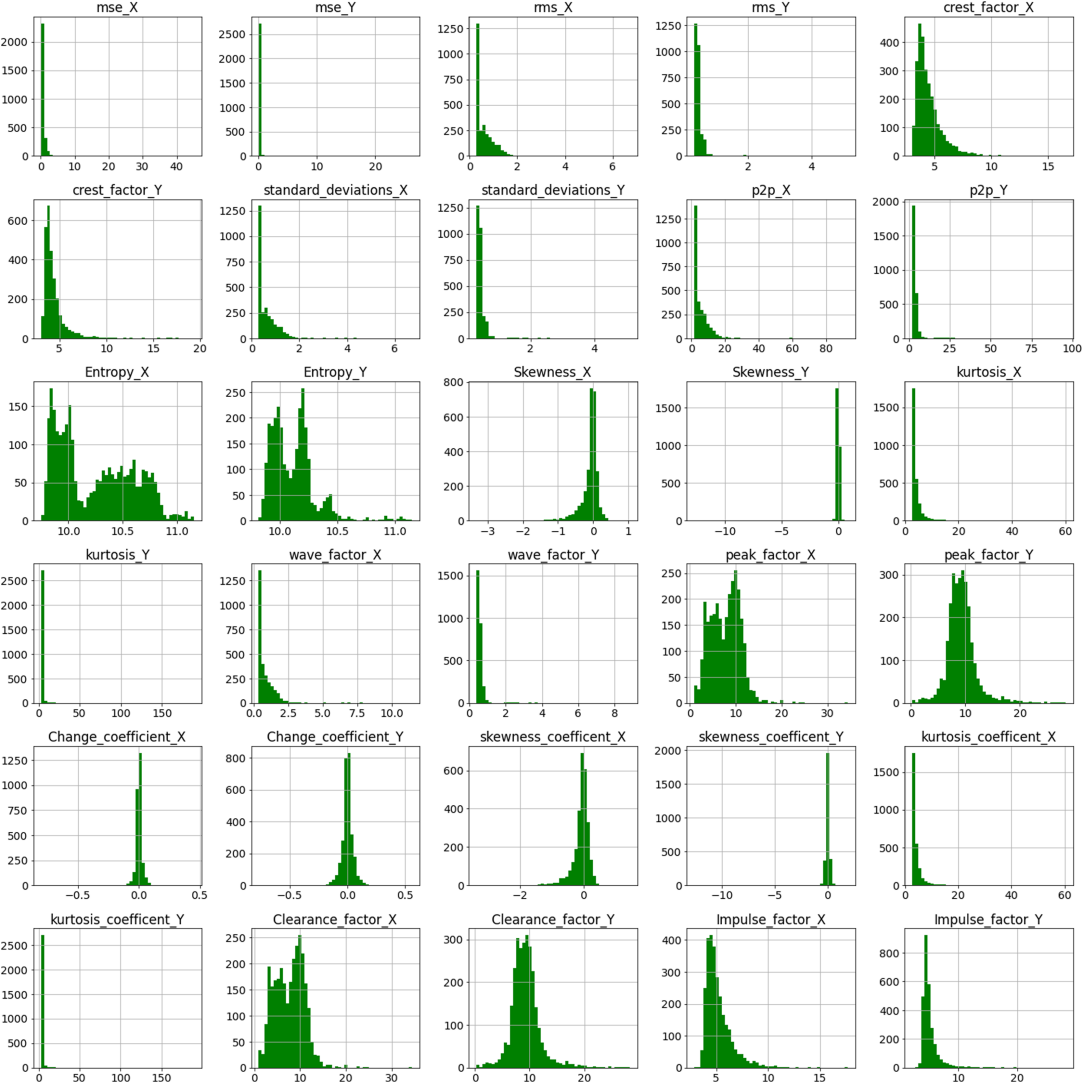
Histogram plot for extracted features of Bearing 1.

Before proceeding to step 2 of Feature ranking and selection, exploratory data analysis (EDA) of the extracted time-domain features was done. Data scientists utilize EDA to examine and investigate data sets and describe their major features, typically using data visualization techniques. Fig. 6 represents the histogram plots for the various extracted time-domain features of Bearing 1. A histogram is a graph that shows the distribution of values for a numeric variable as a set of bars. A bar’s height shows the frequency of data points having a value within the associated bin; each bar generally spans a range of numeric values called a bin or class. The values of features such as crest_factor_X, crest_factor_Y, Entropy_X, Entropy_Y, peak_factor_X, peak_factor_Y, clearance_factor_X and clearance_factor_Y are well distributed over a range of values. However values of mse_X,mse_Y, kurtosis_X, kurtosis_Y, kurtosis_coefficient_X and kurtosis_coefficient_Y mostly lie in the range of 0 to 1. The height of the bar represents the frequency of the value for, eg, for crest_factor_Y, maximum values lie in the range of 0 to 5.

**Figure 7:**
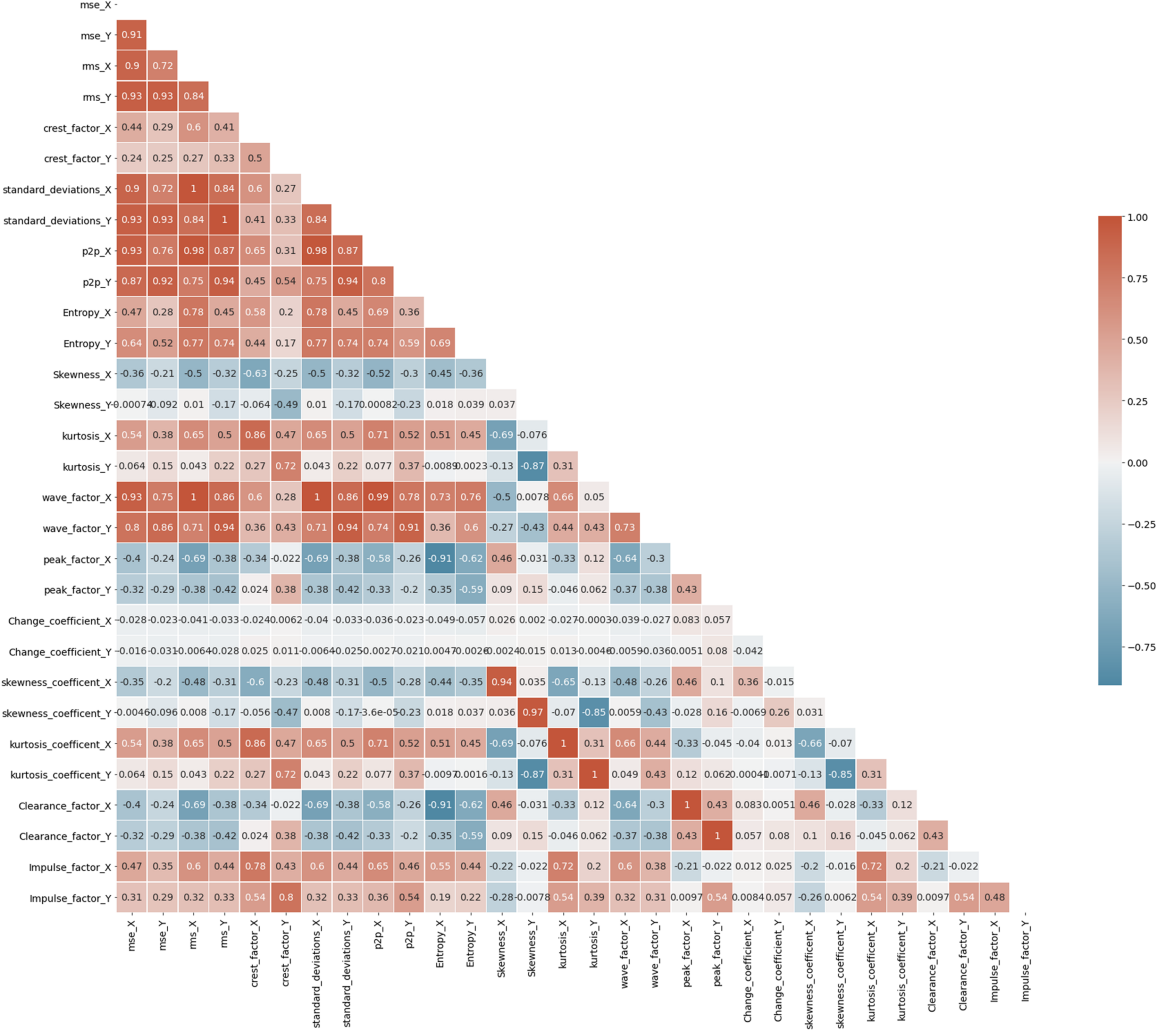
Correlation map of Bearing 1.

A correlation heatmap is a two-dimensional correlation matrix with colored pixels indicating data on a monochrome scale in two discrete dimensions. The values of the first dimension appear as rows in the table, while the values of the second dimension appear as columns. The color of the cell is determined by the number of measurements that match the dimensional value. Correlation heatmaps are useful for data analysis because they reveal differences and variance in the same data while highlighting trends. In a correlation heatmap, like in a traditional heatmap, a color bar improves the reading and interpretation of data. Fig. 7 depicts the correlation matrix for all the features in Bearing 1. The correlation coefficient might have any value between –1, and 1 wherein value towards +1 represents positive correlation, value towards –1 represents negative correlation, and value of 0 represents no correlation. For example, the values of standard_deviation_X and rms_X have values +1, showing positive correlation, and so on. Correlation maps help us analyze the correlation between the various features in a dataset (Pourhomayoun & Shakibi, 2021).

### Feature ranking and feature selection

**Figure 8:**
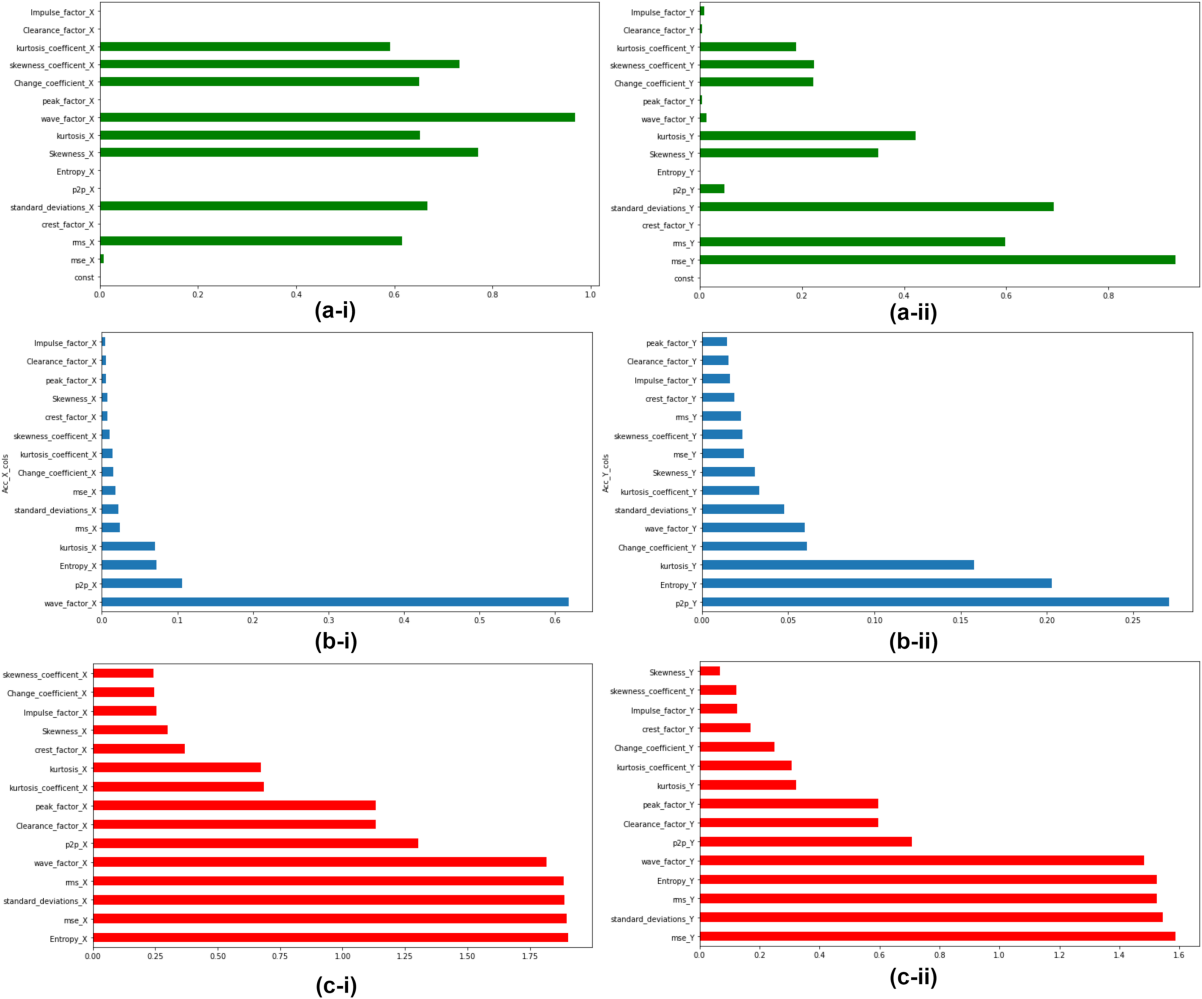
Feature importance for Bearing 1 features as per feature ranking techniques. (A-i & A-ii) Linear regressor for accelerometer X & Y. (B-i & B-ii) Random Forest regressor for accelerometer X & Y. (C-i & C-ii) Mutual Info regressor for accelerometer X & Y.

The extracted features are ranked according to their feature importance as per three feature ranking techniques explained in Section “Linear Regressor”, “Random Forest Regressor”, and “Mutual Info Regressor”. At the end of this step top, five features for accelerometer X and Y respectively were selected for the next stage of unsupervised clustering. Feature Ranking and selection were made on all seven bearings. Fig. 8 depicts the Feature Ranking graphs for Bearing 1. Figs. 8A-i and 8A-ii show the feature ranking graph for accelerometer X and Y using Linear Regressor. Figs. 8B-i and 8B-ii show the feature ranking graph for Accelerometer X and Y using Random Forest Regressor. Figs. 8C-i and 8C-ii show the feature ranking graph for Accelerometer X and Y using Mutual Info Regressor. The authors carried out similar kind of feature ranking and feature selction on all the seven bearings.

### Unsupervised clustering for generation of fault diagnosis data

**Figure 9:**
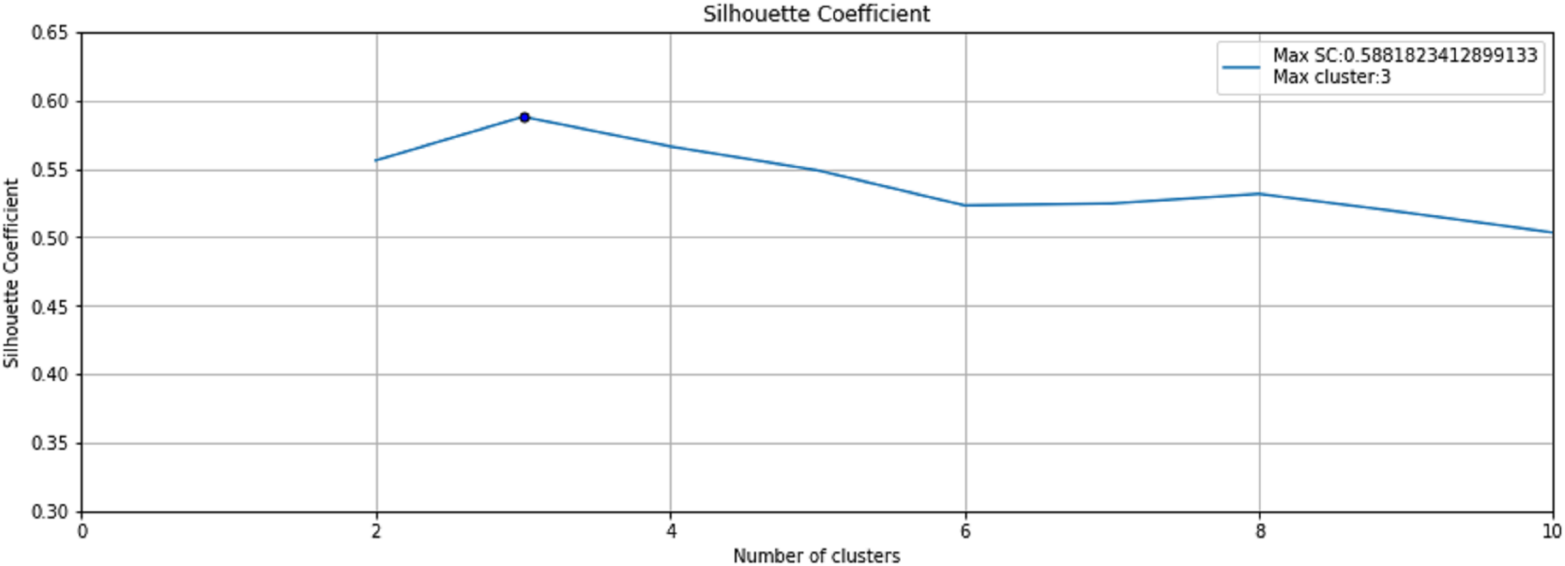
Silhouette Coefficient for Bearing 1.

The step 3 of the Anomaly-Onset Aware framework is the application of an unsupervised K-means algorithm with Silhouette Coefficient on the selected bearing feature data. K-means algorithm helps create similar and dissimilar clusters on basis of Euclidean distance and the Silhouette Coefficient helps in optimal cluster mining. Fig. 9 depicts the Silhouette Coefficient value for Bearing 1. Here SC = 0.588 and Max Cluster value = 3.

**Figure 10:**
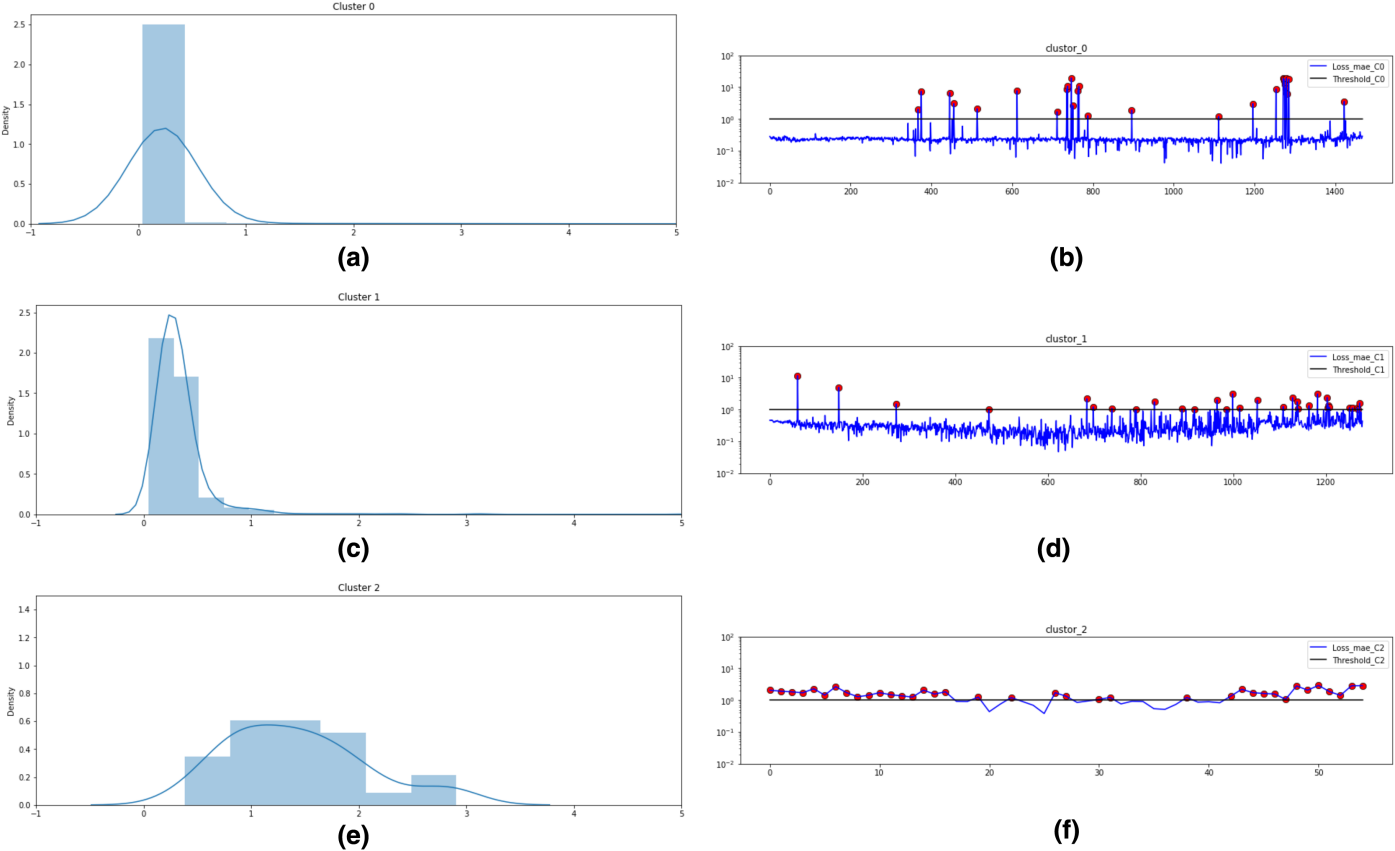
k-means technique for unsupervised clustering on Bearing 1. (A) Threshold = 1.0 for Cluster 0. (B) Abnormal values in Cluster 0. (C) Threshold = 1.0 for Cluster 1. (D) Abnormal values in Cluster 1. (E) Threshold = 2.75 for Cluster 2. (F) Abnormal values in Cluster 2.

Based on these values, as shown in Fig. 10, three clusters are formed for Bearing 1 feature data using the k-means algorithm. Figs. 10A, 10C and 10E plots the probability distribution graph for thresholds in Cluster 0,1 and 2 respectively. Optimal thresholding helps in creating normal and abnormal classes for all three clusters. Figs. 10B, 10D and 1F depict the abnormal values in cluster 0, 1 and 2 respectively. The red points denote the abnormal values in these clusters beyond the cluster threshold. The normal classes and abnormal classes for all the three clusters are merged to create two final clusters-normal and abnormal clusters. These clusters are then sent to Step 4 of the framework to apply semi-supervised anomaly detection.

The above technique is carried out on all seven bearings. The Silhouette Coefficient value and Max Clusters value for all seven bearings is shown in Table 4 below:

**Table 4:** Silhouette coefficient and max clusters value for all seven bearings.

Bearing_No	1	2	3	4	5	6	7
**Silhouette Coefficient value**	0.588	0.780	0.648	0.814	0.649	0.856	0.895
**Max_Clusters value**	3	2	3	2	2	2	2

### Semi-supervised anomaly detection for fault diagnosis

**Figure 11:**
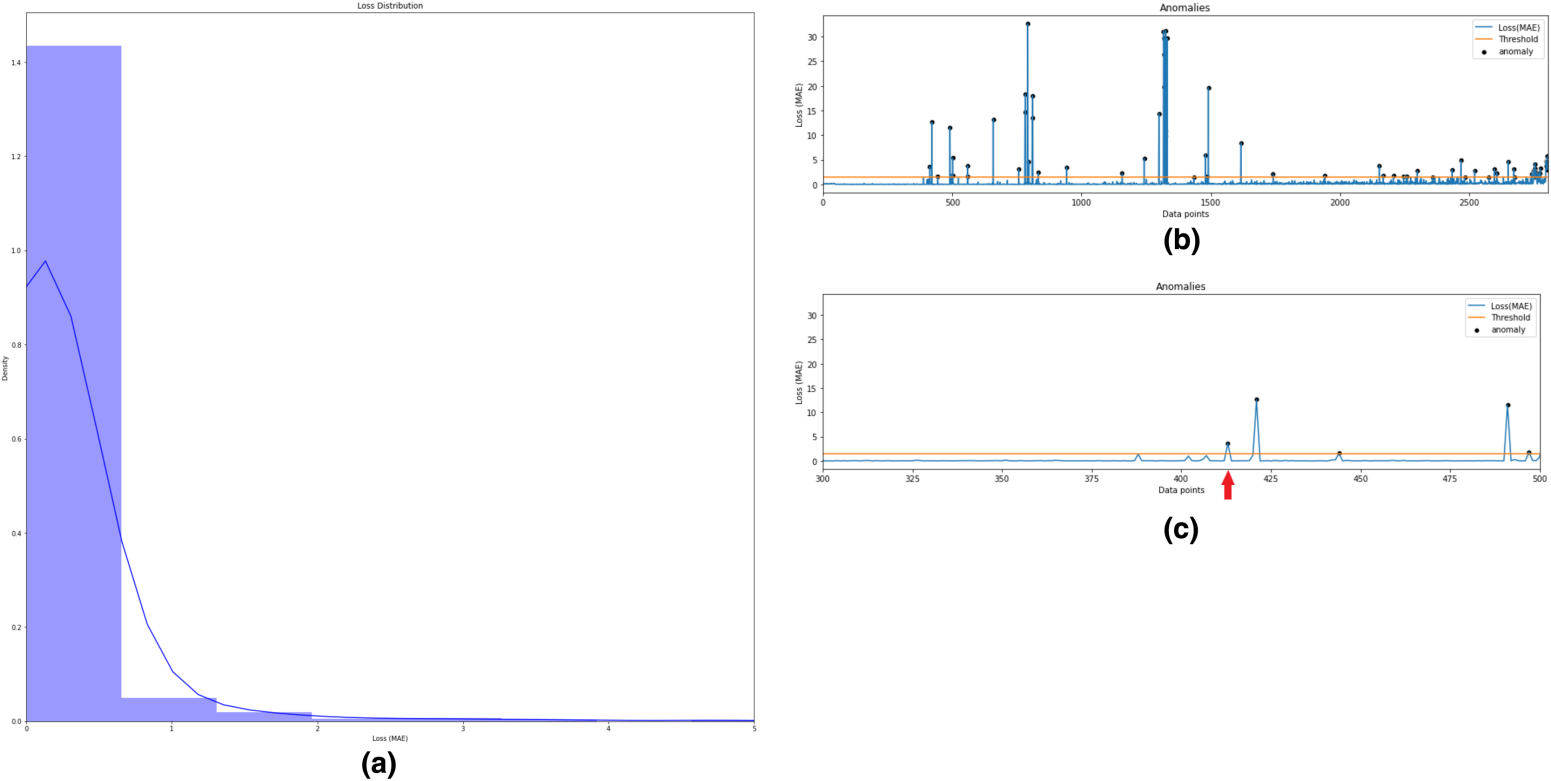
Semi-supervised anomaly detection for Bearing 1. (A) AE-LSTM Reconstruction loss histogram for threshold in Bearing 1. (B) Anomalies detected in test set of Bearing 1. (C) Timestamp for Anomaly-triggered RUL in Bearing 1.

In this step, semi-supervised Autoencoder-LSTM (AE-LSTM) technique is applied on both normal and abnormal clusters generated from Step 3 described above. Training and test sets are split into both clusters. The 80:20 split is used to train the AE-LSTM model on the training set and verify it on the test set. The intelligent neural network automatically assigns weights to the nodes during training and calculates reconstruction loss at each step. Similar to step 3, thresholding on the basis of the reconstruction loss helps identify anomalies in the bearing feature data. Also, the technique helps to indicates the timestamp for the occurrence of the first anomaly. Fig. 11 depicts this process for Bearing 1. Image (a) describes the histogram plot for the AE-LSTM reconstruction loss. The optimal threshold for flagging anomaly points can be identified with the help of this histogram. Image (b) depicts the anomalies highlighted as black points in the test set. Image (c) helps in identifying the first anomaly timestamp. Similar results were obtained for the rest seven bearings.

### Anomaly-triggered RUL prediction for fault prognosis

**Figure 12:**
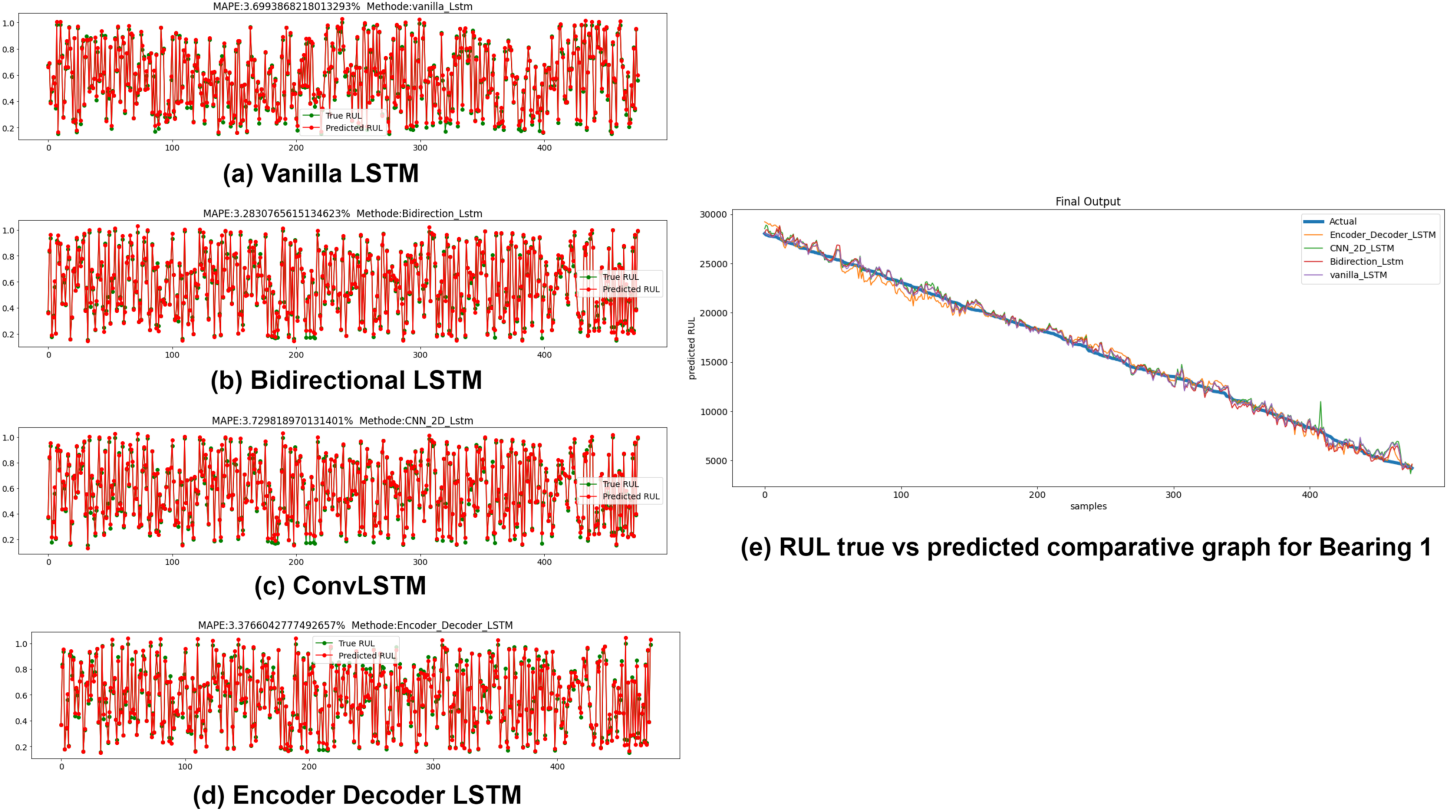
Anomaly triggered RUL prediction using LSTM variants for Bearing 1. (A) Vanilla LSTM. (B) Bidirectional LSTM. (C) Conv LSTM. (D) Encoder-Decoder LSTM. (E) RUL true *vs*. predicted comparative graph for Bearing 1.

The Anomaly-triggered RUL prediction results using the variants of LSTM for all seven bearings are presented in this sub-section. The timestamp of the first anomaly incidence obtained from the previous step of semi-supervised anomaly detection is used to clip the data and only the dataset after that timestamp is fed to the RUL estimators. RUL estimators used in this study are Vanilla LSTM, Bidirectional LSTM, ConvLSTM, and Encoder-Decoder LSTM. Mean squared error (MSE) and Mean Absolute Percentage error (MAPE) are calculated for each variant. Lower the MSE and MAPE values, better is the forecasting accuracy of the model. A comparative graph of true RUL, i.e., actual RUL *vs* predicted RUL of the estimators, is plotted at the end. Fig. 12 depict the MAPE values between True RUL and Predicted RUL for Bearing 1 in case of each LSTM variants: Part (a) Vanilla LSTM, Part (b) Bidirectional LSTM, Part (c) ConvLSTM and Part (d) Encoder Decoder LSTM. Part (e) of Fig. 12 displays the RUL regression comparative graph for all estimators with True RUL. Similar results were obtained for the rest seven bearings.

## Discussion

This section presents a comparative analysis of the anomaly detection and RUL prediction experimental results presented above.

### Comparative analysis of anomaly detection stage

**Figure 13:**
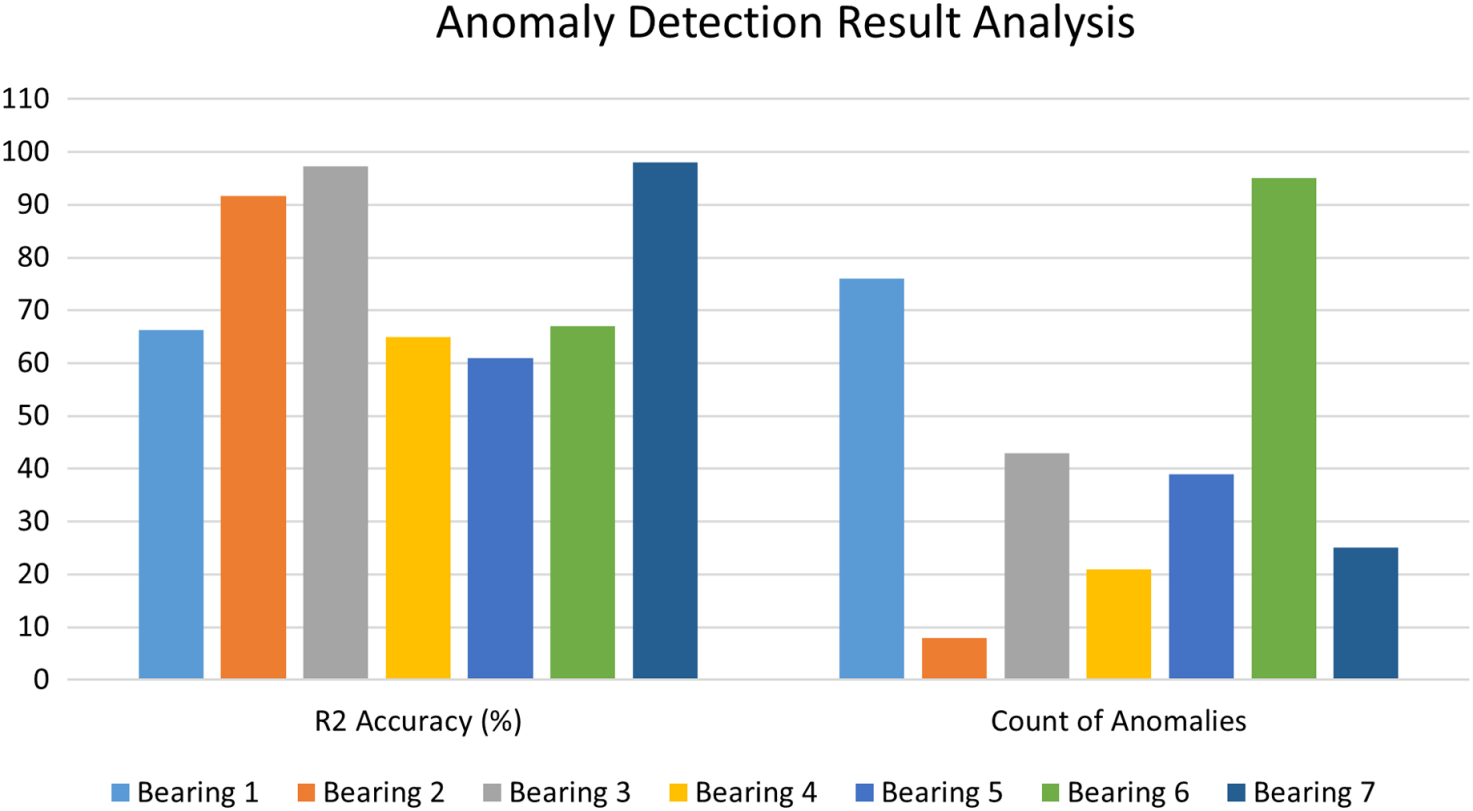
Performance analysis of anomaly detection stage.

The Autoencoder-LSTM accuracy is calculated in terms of R2 accuracy for each of the bearings. The R2 accuracy is also known as the coefficient of determination or goodness of fit. The degree of variance in the output dependent characteristic can be predicted based on the input independent variable(s). It’s used to assess how effectively the model reproduces observed findings based on the ratio of total deviation of results represented by the model. Its value lies between 0 to 100%. Fig. 13 depicts the comparative bar graph of the performance analysis for the Anomaly Detection stage of the proposed framework for all seven bearings in terms of R2 accuracy and the count of anomalies detected for each bearing. In this study, all seven bearings exhibited good R2 scores in the range of 60 to 100%.

### Comparative analysis of anomaly triggered RUL estimation stage

**Figure 14:**
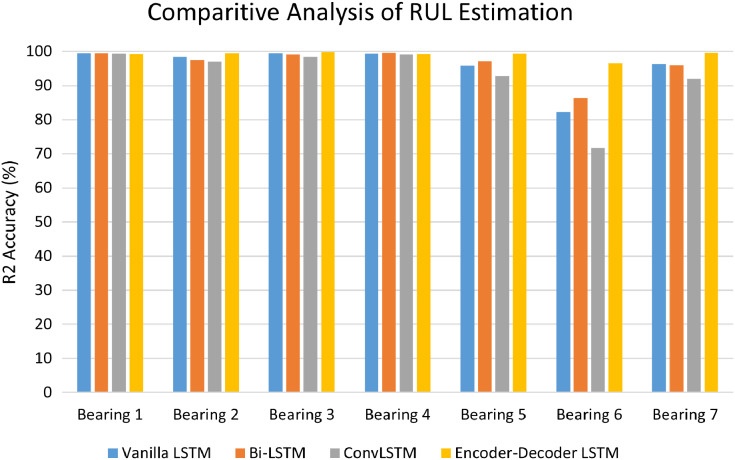
Performance analysis of RUL estimation using LSTM variants.

The performance of LSTM variants for RUL estimation for all the seven bearings was evaluated in terms of R2 accuracy and Mean Square Error (MSE). Fig. 14 depicts the comparative analysis of the R2 accuracy score of the LSTM variants-Vanilla LSTM, Bidirectional LSTM, ConvLSTM, and Encoder-Decoder LSTM for Bearing 1–7. Except for Bearing 6, the variants achieved very high R2 accuracy above 90%. The Encoder-Decoder-LSTM variant exhibited high accuracy consistently across all seven bearings.

Table 5 shows the achieved Mean Square Error values of the LSTM variants used for the Anomaly-triggered remaining useful life estimation of the PRONOSTIA bearings data. The average of error squares, *i.e*. the average squared variation between the estimated and real values, is measured by an estimator’s Mean Squared Error (MSE). It’s a risk factor that represents the squared error loss’s predicted value. It is never negative; therefore, values near 0 are preferable. Lower the error loss, better is the estimator performance, and higher is R2 accuracy. All the LSTM variants measured low MSE’s for RUL estimation in the case of all seven bearings.

**Table 5:** Mean square error-values for RUL estimation using LSTM variants.

MSE	Bearing 1	Bearing 2	Bearing 3	Bearing 4	Bearing 5	Bearing 6	Bearing 7
**Vanilla LSTM**	0.00030	0.00129	0.00047	0.00039	0.00340	0.01577	0.00303
**Bi-LSTM**	0.00033	0.00196	0.00072	0.00023	0.00237	0.01211	0.00336
**ConvLSTM**	0.00041	0.00235	0.00137	0.00063	0.00588	0.02512	0.00654
**Encoder-Decoder LSTM**	0.00045	0.00038	0.00014	0.00049	0.00050	0.00302	0.00035

## Conclusions

The main focus of this study is the development of a combined Anomaly Detection-RUL prediction framework in the case of rotating machinery such as bearings. A unique anomaly-onset aware remaining usable life (AOA-RUL) estimating technique is presented to avoid the complexities and performance degradations associated with machine learning-based RUL estimators when RULs are ill-defined during normal operation. The AOA-RUL uses the k-means algorithm with Silhouette Coefficient for unsupervised clustering of unannotated vibration data, which is the case in most real-time monitoring use-cases. The framework uses effective deep learning models such as Autoencoders and LSTM which exhibit intelligent capabilities in prediction over longer sequences. Across the samples, the AOA-RUL technique improves RUL estimation with less computational overhead, suggesting increased resilience. The contributions of this study are:

- Development of clustering technique for anomaly pattern analysis on unlabeled data.
- Combined deep-learning-based anomaly triggered RUL estimation technique using hybrid semi-supervised Autoencoder-LSTM for anomaly detection having accuracy in the range of 60 to 90% across various bearings.
- LSTM based RUL estimators having prediction accuracy of over 90% for variants of Vanilla LSTM, Bidirectional LSTM, ConvLSTM, and Encoder-Decoder LSTM.

The current framework is restricted to non-varying operational scenarios. The suggested AOA-RUL scheme is general. It may be used with a variety of anomaly detectors and DL-based RUL estimators, such as one that is suitable for varied operating circumstances. Such variations are outside the scope of this study and will be addressed in the future using the transfer learning approach.

## Additional Information and Declarations

### Data Availability

The following information was supplied regarding data availability: The code is available at GitHub: https://github.com/PoojaPaiKamat/AnomalyOnsetAwareRUL. The Pronostia IEEE 2012 bearing dataset (Femto dataset) is available at NASA: “FEMTO Bearing Data Set”, NASA Ames Prognostics Data Repository (http://ti.arc.nasa.gov/project/prognostic-data-repository), NASA Ames Research Center, Moffett Field, CA. https://ti.arc.nasa.gov/tech/dash/groups/pcoe/prognostic-data-repository/.
